# Synthesis and in vitro antitumor activity of (1*E*,4*E*)-1-aryl-5-(2-((quinazolin-4-yl)oxy)phenyl)-1,4-pentadien-3-one derivatives

**DOI:** 10.1186/s13065-017-0253-9

**Published:** 2017-03-15

**Authors:** Hui Luo, Shengjie Yang, Da Hong, Wei Xue, Pu Xie

**Affiliations:** 1 Guizhou Fruit Institute, Guizhou Academy of Agricultural Sciences, Guiyang, 550006 P. R. China; 2 R&D Center, Sinphar Tian-Li Pharmaceutical Co., Ltd, Hangzhou, 311100 P. R. China; 3 State Key Laboratory Breeding Base of Green Pesticide and Agricultural Bioengineering, Key Laboratory of Green Pesticide and Agricultural Bioengineering, Ministry of Education, Guizhou University, Guiyang, 550025 P. R. China; 4 Ctr for R&D of Fine Chemicals, Guizhou University, Guiyang, 550025 P. R. China

**Keywords:** Synthesis, Asymmetric curcumin analogs, Quinazoline derivatives of curcumin, Antitumor activity, MGC-803, Apoptosis

## Abstract

**Background:**

Cancer is one of the leading causes of death and only second to heart diseases. Recently, preclinical studies have demonstrated that curcumin had a number of anticancer properties. Thus, we planned to synthesize a series of curcumin analogs to assess their antiproliferation efficacy.

**Results:**

A series of (1*E*,4*E*)-1-aryl-5-(2-((quinazolin-4-yl)oxy)phenyl)-1,4-pentadien-3-one derivatives (curcumin analogs) were synthesized and characterized by IR, NMR, and elemental analysis techniques. All of the prepared compounds were screened for antitumor activities against MGC-803, PC3, and Bcap-37 cancer cell lines. A significant inhibition for cancer cells were observed with compound **5f** and also less toxic on NIH3T3 normal cells. The mechanism of cell death induced by compound **5f** was further investigated by acridine orange/ethidium bromide staining, Hoechst 33,258 staining, TUNEL assay, and flow cytometry cytometry, which revealed that the compound can induce cell apoptosis in MGC-803 cells.

**Conclusions:**

This study suggests that most of the derivatives could inhibit the growth of human cancer cell lines. In addition, compound **5f** could induce apoptosis of cancer cells, and it should be subjected to further investigation as a potential anticancer drug candidate.

## Background

Cancer is one of the leading causes of death and only second to heart diseases [1, 2]. The efficacy of current chemotherapeutics is low and undesirable side effects are still unacceptably high [3–5]. Hence, the development of novel, and less toxic and anti-cancer agents remains an important and challenging goal of medicinal chemist worldwide, and much attention has recently been paid to the discovery and development of new, more selective anticancer agents [3, 6–8].

Natural products have become a leading category of compounds in improving the rational drug design for novel anti-cancer therapeutics [9, 10]. Curcumin is a natural phenolic compound originally isolated from turmeric, a rhizome used in India for centuries as a spice and medicinal agent [11]. A literature survey reveals that curcumin, and its derivatives (analogs) have various pharmacological activities and medicinal applications such as antioxidant [12, 13], anti-inflammatory [12, 14], anti-HIV [15, 16], anti-angiogenesis and so on [12]. Recently, preclinical studies have demonstrated that curcumin had a number of anticancer properties, such as growth inhibition and induction of apoptosis in a variety of cancer cell lines [17–19]. Its mechanisms of action include inhibition of transcriptional factor NF-jB, HSP90 and epigenetic modulation related to direct inhibition of the catalytic site of DNMT-1 [20]. Moreover, the latest research shows that curcumin can effectively suppress NF-kB activity and COX-2 expression, as well as cell proliferation/survival in the setting of NSCLC [21]. Consequently, analogues of curcumin with similar safety profiles but increased anticancer activity have been developed in recent years [22]. Chandru et al. synthesized four novel dienone cyclopropoxy curcumin analogs by nucleophilic substitution reaction with cyclopropyl bromide, and found that the tumor growth inhibitory effects of synthetic dienone cyclopropoxy curcumin analogs could be mediated by promoting apoptosis and inhibiting tumor angiogenesis [23]. New 1,5-diaryl-1,4-pentadien-3-one derivatives (curcumin analogs), which can effectively inhibit proliferation of cancer cells at very low concentrations, were synthesized [24, 25], and we also found that curcumin analogs exhibited promising ex vivo antiviral bioactivities against tobacco mosaic virus and cucumber mosaic virus [26].

**Fig. 1:**
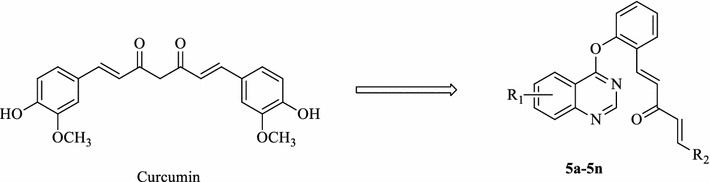
Design of the target compounds

In order to discover more potent and selective anticancer agents based on curcumin scafforld, we have synthesized a series of (1*E*,4*E*)-1-aryl-5-(2-((quinazolin-4-yl)oxy)phenyl)-1,4-pentadien-3-one derivatives (eleven novel compounds **5a**, **5b**, **5d**, **5f**–**5h**, and **5j**–**5n**) (Fig. 1). In our present study, all the target compounds were evaluated for their activity against MGC-803, PC3, and Bcap-37 cancer cell lines. Furthermore, the possible mechanism of MGC-803 cell growth inhibition by compound **5f** was also investigated in this paper.

## Results and discussion

### Chemistry

**Scheme 1:**
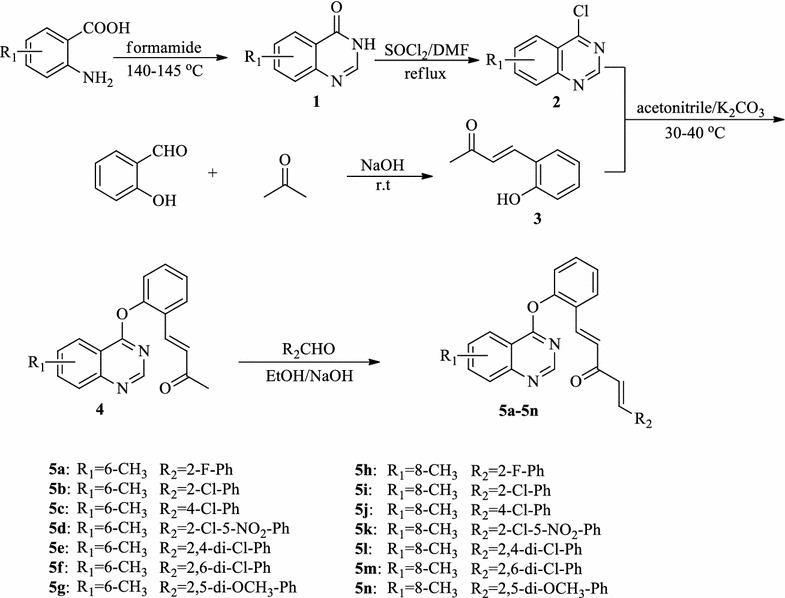
Synthetic pathway to target compounds **5a**–**5n**

Target compounds **5a**–**5n** were synthesized as shown in Scheme 1. The starting material 2-aminobenzoic acid was conveniently cyclized to intermediate **1** by heating it with formamide at 140–145 °C as described in the literature. Upon refluxing with freshly distilled phosphorus oxychloride and pentachlorophosphorane, intermediate **1** yielded the corresponding 4-chloro derivative **2**. Treatment of salicylaldehyde with acetone in the presence of sodium hydride at room temperature got intermediate **3**. The key intermediates **4** were synthesized by reacting intermediate **3** with substituted 4-chloroquiazoline **2** in the present of K_2_CO_3_ in CH_3_CN at 30–50 °C for 6 h. And then, the target compounds **5a**–**5n** were synthesized by reacting the substituted aldehydes with **4** in the present of anhydrous alcohol in acetone at room temperature. The structures of the final products were confirmed by their IR, ^1^H NMR, ^13^C NMR, and elemental analysis techniques.

### Evaluation of anti-tumor bioactivity of synthetic compounds

The in vitro antitumor activity of the newly synthesized compounds **5a**–**5n** were evaluated against a panel of three human cancer cell lines, including human gastric cancer cell line MGC-803, human prostate cancer cell line PC3, and human breast cancer cell line Bcap-37, and one normal cell line NIH3T3 (mouse embryo fibroblast cell line) by MTT method. Adriamycin (ADM) was chosen as a reference drug due to its availability and widespread use. Each experiment was repeated at least three times. The results are presented in Table 1.

**Table 1:** Effect of title compounds against cell viability of different cell lines

Compds	Inhibition rates against different cells (%)^a^				IC_50_ (μM)^b^		
	MGC-803	PC3	Bcap-37	NIH3T3	MGC-803	PC3	Bcap-37
**5a**	71.9 ± 0.9	68.5 ± 2.3	44.1 ± 2.6	65.7 ± 1.3	7.69 ± 0.37	9.24 ± 0.46	11.71 ± 0.39
**5b**	83.5 ± 1.5	78.7 ± 2.4	57.3 ± 2.0	15.4 ± 1.5	5.20 ± 0.51	8.12 ± 0.46	10.05 ± 0.67
**5c**	79.2 ± 2.8	76.5 ± 1.7	54.2 ± 1.1	16.1 ± 2.7	5.44 ± 0.45	7.85 ± 0.32	9.34 ± 0.52
**5d**	87.5 ± 0.2	86.3 ± 0.8	58.5 ± 1.3	71.9 ± 1.4	1.72 ± 0.22	2.68 ± 0.43	7.52 ± 0.36
**5e**	87.0 ± 1.0	75.2 ± 7.8	67.3 ± 3.9	53.8 ± 1.8	1.89 ± 0.26	4.71 ± 0.36	9.6 ± 0.42
**5f**	90.7 ± 0.6	93.0 ± 1.8	76.5 ± 1.9	21.5 ± 2.7	0.85 ± 0.43	1.37 ± 0.22	4.98 ± 0.39
**5g**	85.9 ± 1.2	83.1 ± 2.8	74.9 ± 2.0	74.3 ± 0.9	2.02 ± 0.37	2.18 ± 0.31	5.61 ± 0.50
**5h**	62.3 ± 1.4	43.7 ± 12.1	36.6 ± 2.7	13.4 ± 2.2	9.06 ± 0.40	11.68 ± 0.48	15.64 ± 0.51
**5i**	79.4 ± 2.4	65.1 ± 2.6	47.1 ± 2.6	14.4 ± 2.6	7.78 ± 0.48	9.36 ± 0.52	10.73 ± 0.62
**5j**	76.3 ± 0.8	71.9 ± 3.1	45.4 ± 2.2	41.0 ± 2.1	6.42 ± 0.44	8.64 ± 0.47	12.11 ± 0.31
**5k**	82.1 ± 3.0	76.3 ± 2.5	60.2 ± 1.4	3.0 ± 2.3	2.94 ± 0.32	8.12 ± 0.46	8.92 ± 0.58
**5l**	61.2 ± 1.3	55.7 ± 1.5	54.6 ± 1.0	0.8 ± 1.6	4.33 ± 0.35	9.08 ± 0.52	9.83 ± 0.46
**5m**	81.1 ± 1.7	81.2 ± 2.8	66.4 ± 1.5	12.4 ± 2.5	2.05 ± 0.39	3.79 ± 0.47	7.82 ± 0.39
**5n**	78.7 ± 2.7	69.0 ± 1.7	55.8 ± 1.1	29.6 ± 2.4	3.33 ± 0.42	4.16 ± 0.43	8.70 ± 0.54
ADM^c^	97.5 ± 1.2	91.2 ± 0.4	94.5 ± 0.9	100.0 ± 0.7	0.74 ± 0.24	1.01 ± 0.20	1.90 ± 0.25

^a^Inhibitory percentages of cells treated with 10 μM concentration of each compound for 72 h

^b^Agent concentration (μM) that inhibited cell growth by 50% at 72 h after treatment

^c^Adriamycin, positive control

As depicted in Table 1, the title compounds suppressed proliferation of the above three cancer cell lines in different extents (IC_50_ values of 0.85–15.64 μM), and exhibited broad spectrum antitumor activity. Among these studied compounds, the inhibitory ratios of **5d**–**5g**, and **5m** against MGC-803 cells at 10 μM were 87.5, 87.0, 90.7, 85.9, and 81.1%, respectively, and their IC_50_ values were 1.72, 1.89, 0.85, 2.02, and 2.05 μM, respectively, similar to that of ADM (0.74 μM). Compounds **5d**, **5f**, **5g**, and **5m** displayed higher inhibitory activities against PC3 cells at 10 μM than that of the rest compounds, with inhibitory ratios of 86.3, 93.0, 83.1, and 81.2%, respectively, which were similar to or higher than that of ADM (91.2%). The inhibitory ratios of **5f** and **5g** against Bcap-37 cells at 10 μM, were 76.5 and 74.9% (IC_50_ values of 4.98 and 5.61 μM), respectively, which were higher than that of the rest compounds. Also noteworthy is that the potency of the compounds was generally more pronounced against the MGC-803 cells than against PC3 and Bcap-37 cells. Moreover, the antiproliferation activities of the title compounds against NIH3T3 normal cell line were also evaluated. Most of the title compounds showed stronger antiproliferative activities against the cancer cell lines than NIH3T3 lines. Compound **5f**, which showed excellent levels of inhibition against MGC-803, PC3, and Bcap-37 cancer cells, have no significant activity against NIH3T3 cells, with inhibitory ratio of 21.5% at 10 μM. That is to say that the compound was less toxic on normal fibroblasts than on the investigated cancer cell lines and more selective to cancer cells.

Subsequently structure–activity relationships (SAR) studies were performed to determine how the substituents affected the anticancer activity. To examine SAR, different substituent groups were introduced into R_1_ and R_2_ in the quiazoline ring. Based on the activity values indicated in Table 1, the relationships of the activities with different R_1_ and R_2_ (type, position, and number of substituents) were deduced. Two main conclusions were drawn. On the one hand, compared with the same substituents on quiazoline, the corresponding molecules containing a 6-methyl group always had higher inhibitory rates than the compound containing a 8-methyl group. For example, the IC_50_ values of **5f** (R_1_: 6-methyl, R_2_: 2,6-dichlorophenyl) and **5m** (R_1_: 8-methyl, R_2_: 2,6-dichlorophenyl) on MGC-803 cells were 0.85 and 2.05 μM, respectively. By contrast, the inhibition rates of **5c** (R_1_: 6-methyl, R_2_: *p*-chlorophenyl) and **5j** (R_1_: 8-methyl, R_2_: *p*-chlorophenyl) at 10 μM were 79.2 and 76.3% on MGC-803 cells, 76.5 and 71.9% on PC3 cells, and 54.2 and 45.4% on Bcap-37 cells, respectively. On the other hand, when R_2_ was *o*-flurophenyl-fixed, the compounds always showed weak activity. For example, the inhibition rates of **5a** (R_1_: 6-methyl, R_2_: *o*-flurophenyl) at 10 μM were 71.9, 68.5, and 44.1% on the three cancer cells, respectively, which suggested the weaker activity than that of the rest compounds.

Apoptosis is one of the major pathways that lead to the process of cell death [27]. Most cancer cells retain their sensitivity to some apoptotic stimuli from chemotherapeutic agent [28]. In the present study, compound **5f** was selected and its mechanism of growth inhibition of MGC-803 cells was evaluated. To determine whether antiproliferation and cell death are associated with apoptosis, MGC-803 cells were stained with acridine orange (AO)/ethidium bromide (EB) staining and Hoechst 33,258 staining after exposure to compound **5f** and observed under fluorescence microscopy.

It is well known that AO can pass through cell membranes, but EB cannot. Under the fluorescence microscope, living cells appear green. Necrotic cells stain red but have a nuclear morphology resembling that of viable cells. Apoptotic cells appear green, and morphological changes such as cell blebbing and formation of apoptotic bodies will be observed [29].

**Fig. 2:**
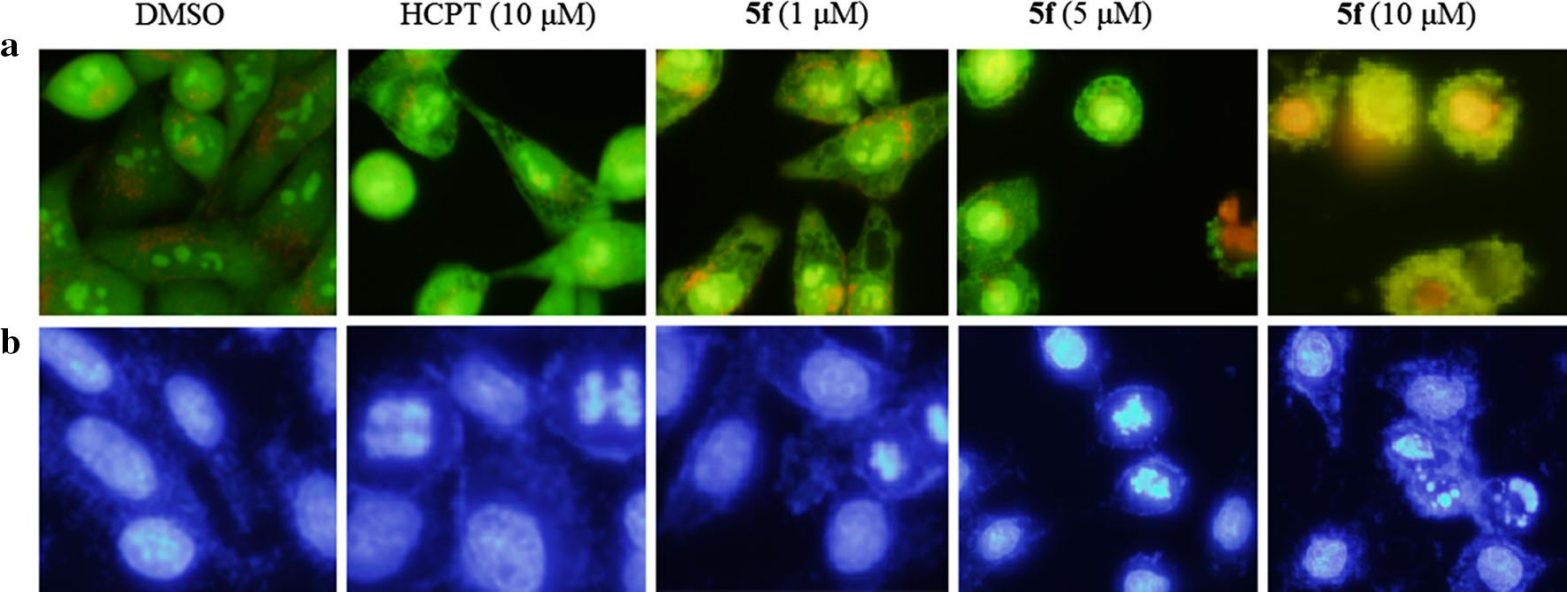
Apoptosis induction studies of compound **5f**. **a** AO\EB staining. **b** Hoechst 33,258 staining

Representative images of the cells treated with 10 μM of HCPT (used as positive control) and 1, 5, 10 μM of compound **5f** for 12 h are shown in Fig. 2a. While treatment of cells with HCPT and compound **5f**, the apoptotic cells with typical apoptotic features, such as staining brightly, condense chromatin and fragment nuclei were observed. These results suggested that the proliferative inhibition and the death of target cells upon treatment with compound **5f** were consequent to the induction of apoptosis.

Membrane-permeable Hoechst 33,258 was a blue fluorescent dye and stained the cell nucleus. When cells were treated with Hoechst 33,258, live cells with uniformly light blue nuclei were observed under fluorescence microscope, while apoptotic cells exhibited bright blue because of karyopyknosis and chromatin condensation, and the nuclei of dead cells could not be stained [30]. MGC-803 cells treated with compound **5f** at concentrations of 1, 5, and 10 μM for 12 h were stained with Hoechst 33,258, with HCPT as positive control at 10 μM for 12 h. The results are illustrated in Fig. 2b.

Figure 2b shows that MGC-803 cells treated with the negative control DMSO were normally blue. Compared with the negative control, a part of cells with smaller nuclei and condensed staining appeared in the positive control group. After treated with compound **5f**, the cells exhibited strong blue fluorescence and revealed typical apoptotic morphology. These findings demonstrate that compound **5f** induced apoptosis against MGC-803 cell lines, consistent with the results for AO/EB double staining.

**Fig. 3:**
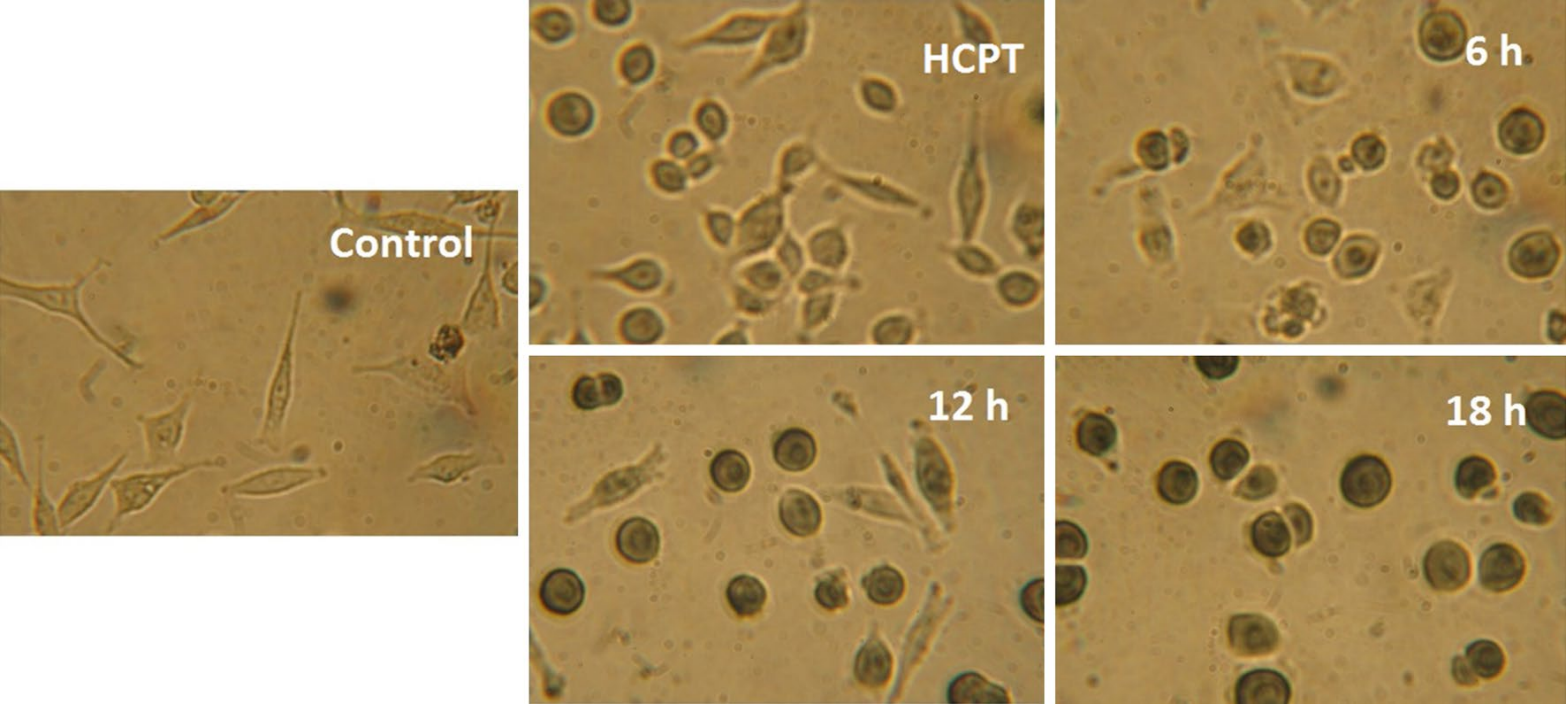
Apoptosis was assayed with TUNEL after treatment of MGC-803 cells with 5 μM **5f**, and observed under light microscopy

To further verify AO/EB and Hoechst 33,258 staining results, TUNEL assay was also carried out. TUNEL (Terminal deoxynucleotidyl Transferase Biotin-dUTP Nick End Labeling) is a very popular assay for identifying apoptotic cells. The assay identifies apoptotic cells in situ by using terminal deoxynucleotidyl transferase (TdT) to transfer biotin-dUTP to these strand breaks of cleaved DNA. The biotin-labeled cleavage sites are then detected by reaction with HRP conjugated streptavidin and visualized by DAB showing brown color [24]. MGC-803 cells treated with compound **5f** at 5 μM for 6, 12, and 18 h were stained with TUNEL, with HCPT as positive control at 5 μM for 18 h. As shown in Fig. 3, cells in control group (DMSO treatment) did not appear as brown precipitates. However, the cells treated with compound **5f** and HCPT appeared as brown precipitate. We further concluded that compound **5f** induced apoptosis against MGC-803.

**Fig. 4:**
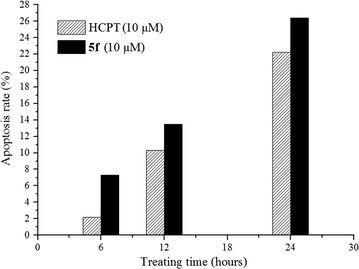
The apoptosis ratios of MGC-803 cells treated with compound **5f** and HCPT

**Fig. 5:**
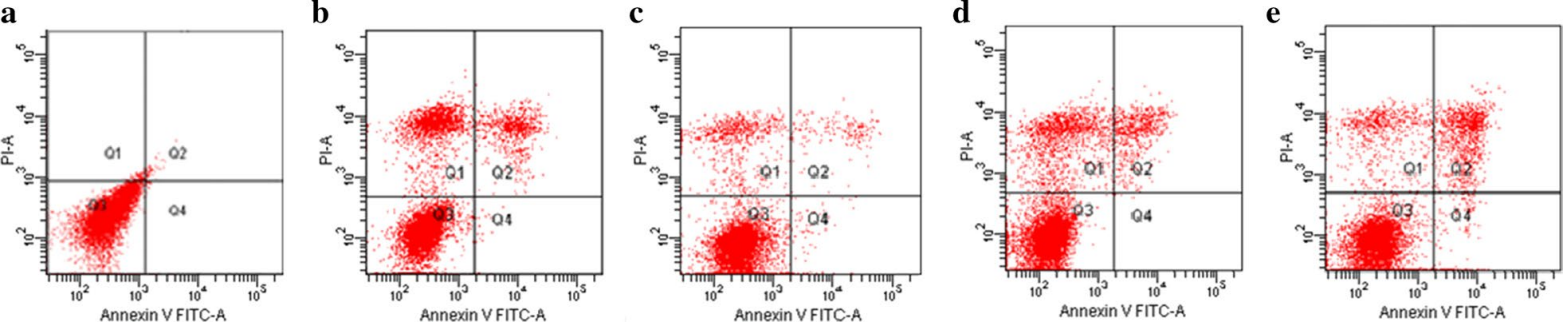
Annexin V/PI dual staining of MGC-803 cell lines. **a** Negative control; **b** treated with HCPT at 10 μM for 24 h; **c**–**e** treated with compound **5f** at 10 μM for 6, 12, and 24 h, respectively

In addition, the apoptosis ratios induced by compound **5f** in MGC-803 cells were determined by flow cytometry, using Annexin V/PI double staining. Flow cytometry was performed on the total cell population (including both adherent and detached cells) and apoptosis detection was carried out as mentioned above. This double staining procedure discriminated necrotic cells (Q1, Annexin^−^/PI^+^), late apoptotic cells (Q2, Annexin^+^/PI^+^), intact cells (Q3, Annexin^−^/PI^−^) and early apoptotic cells (Q4, Annexin^+^/PI^−^) [31, 32]. As shown in Fig. 4, compound **5f** could induce apoptosis of MGC-803 cells, and the highest apoptosis ratio (26.4%) was obtained after 24 h of treatment at a concentration of 10 μM. For the positive control HCPT, the apoptosis ratio was only 22.3% after 24 h of treatment at a concentration of 10 μM. In addition, as shown in Fig. 5, the apoptosis of MGC-803 cells treated with compound **5f** gradually increased in a time-dependent manner.

## Conclusions

As a development of our previous studies, we have synthesized and evaluated in vitro a series of (1*E*,4*E*)-1-aryl-5-(2-((quinazolin-4-yl)oxy)phenyl)-1,4-pentadien-3-one derivatives as potential antitumor agents. Most of the derivatives exhibited equivalent inhibitory activities against MGC-803, PC3, and Bcap-37 cancer cells. Compound **5f** appeared to be more effective than other compounds against the three cells, with IC_50_ values of 0.85, 1.37, and 4.98 μM, respectively. And compounds **5f** was found to exhibit a good degree of selectivity towards cancer cells than normal cells. In addition, the apoptosis-inducing activity of compound **5f** in MGC-803 cells was investigated by AO/EB staining, Hoechst 33,258 staining, TUNEL assay, and flow cytometry. The results revealed that the compound may inhibit cell growth by inducing apoptosis, with apoptosis ratio of 26.4% at 10 μM for 24 h, which was higher than that of HCPT (22.3% at 10 μM for 24 h). Further studies on the specific mechanisms of compound **5f** in MGC-803 cells are currently underway.

## Experimental

### Reagents and chemicals

Melting points were determined by using an XT-4 binocular microscope (Beijing Tech Instrument Co., China) without correction. IR spectra were recorded on a Bruker VECTOR 22 spectrometer. NMR spectra were recorded in a CDCl_3_ solvent using a JEOL-ECX 500 NMR spectrometer operating at 500 MHz for ^1^H, and at 125 MHz for ^13^C by using TMS as internal standard. Elemental analysis was performed on an Elementar Vario-III CHN analyzer. Silica gel (200–300 mesh) and TLC plates (Qingdao Marine Chemistry Co., Qingdao, China) were used for chromatography. All solvents (Yuda Chemistry Co., Guiyang, China) were analytical grade, and used without further purification unless otherwise noted.

### Synthetic procedures

6-methyl-quinazolin-4(3*H*)-one, 8-methyl-quinazolin-4(3*H*)-one, 6-methyl-4-chloroquiazoline, and 8-methyl-4-chloroquiazoline were prepared according to a previously described method [33]. Intermediate (*E*)-4-(2-hydroxyphenyl)-3-butylene-2-one was prepared according to a previously reported [34].

#### General synthetic procedures for the preparation of compounds **5a**–**5n**

Compounds **2** (10 mmol), **3** (10 mmol) and K_2_CO_3_ (70 mmol) in 20 mL of acetonitrile was stirred at 30–40 °C for 3.5 h. The reaction mixture was concentrated and allowed to cool. The solid product obtained was filtered, and recrystallized with ethanol to afford the desired solid compound **4a** or **4b**, respectively. To the mixture of compound 4a or 4b (0.5 mmol) and sodium hydroxide (1%) in 20 mL of 75 vol% ethanol/water solution was added substituted aldehydes (0.5 mmol). The reaction mixture was stirred at room temperature overnight. The reaction mixture was concentrated and suspended in water (20 mL), adjusted with 5% HCl to pH 7, and filtered. Recrystallization with ethanol afforded the desired solid compounds **5a**–**5n**.

##### (1E,4E)-1-(2-fluorophenyl)-5-(2-((6-methylquinazolin-4-yl)oxy)phenyl)penta-1,4-dien-3-one (**5a**)

Yield: 52.6%; yellow powder; mp: 121–123 °C; IR (KBr, cm^−1^) *ν*: 3442, 1657, 1622, 1596, 1465, 1398, 1356, 1221, 983; ^1^H NMR (CDCl_3_, 500 MHz) *δ:* 8.70 (s, 1H, Qu-2-H), 8.23 (d, *J* = 12.00 Hz, 1H, F–Ar–CH=), 7.93 (d, *J* = 8.6 Hz, 1H, Ar–CH=), 7.78–7.85 (m, 3H, Qu-5,7,8-H), 7.47–7.50 (m, 3H, F–Ar-4,6-H, Ar-3-H), 7.30–7.39 (m, 5H, F–Ar-3,5-H, Ar-4,5-H, F–Ar–C=CH), 7.10 (d, *J* = 16.0 Hz, 1H, Ar–C=CH), 6.81 (d, *J* = 14.8 Hz, 1H, Ar-6-H), 2.61 (s, 3H, CH_3_); ^13^C NMR (CDCl_3_, 125 MHz) *δ*: 188.8, 166.4, 153.4, 153.4, 151.7, 150.4, 136.9, 136.7, 136.5, 131.7, 129.4, 128.2, 127.9, 127.7, 127.2, 127.1, 126.6, 126.5, 123.6, 123.5, 122.3, 116.4, 21.9; Anal. Calcd for C_25_H_19_FN_2_O_2_: C 76.08; H 4.67; N 6.83; Found: C 76.42; H 4.78; N 6.80.

##### (1E,4E)-1-(2-chlorophenyl)-5-(2-((6-methylquinazolin-4-yl)oxy)phenyl)penta-1,4-dien-3-one (**5b**)

Yield: 46.3%; yellow powder; mp: 152–154 °C; IR (KBr, cm^−1^) *ν*: 3445, 1653, 1618, 1584, 1481, 1400, 1359, 1223, 986; ^1^H NMR (CDCl_3_, 500 MHz) *δ*: 8.69 (s, 1H, Qu-2-H), 8.22 (d, *J* = 8.0 Hz, 1H, Cl–Ar–CH=), 7.76–7.95 (m, 4H, Ar–CH=, Qu-5,7,8-H), 7.38–7.53 (m, 3H, Cl–Ar-3,6-H, Ar-3-H), 7.23–7.31 (m, 5H, Cl–Ar-4,5-H, Ar-5-H, Ar–C=CH, Cl–Ar–C=CH), 7.21 (m, 1H, Ar-4-H), 6.82 (d, *J* = 14.8 Hz, 1H, Ar-6-H), 2.62 (s, 3H, CH_3_); ^13^C NMR (CDCl_3_, 125 MHz) *δ*: 188.6, 167.1, 154.3, 153.1, 151.0, 142.6, 142.1, 136.5, 134.5, 133.3, 132.6, 130.0, 129.6, 129.4, 127.4, 125.8, 125.6, 122,9, 122.7, 121.2, 116.3, 17.7; Anal. Calcd for C_26_H_19_ClN_2_O_2_: C 73.2; H 4.50; N 6.56; Found: C 73.27; H 4.56; N 6.42.

##### (1E,4E)-1-(4-chlorophenyl)-5-(2-((6-methylquinazolin-4-yl)oxy)phenyl)penta-1,4-dien-3-one (**5c**)

Yield: 55.8%; yellow powder; mp: 173–176 °C; IR (KBr, cm^−1^) *ν*: 3445, 1653, 1622, 1558, 1489, 1373, 1229, 986; ^1^H NMR (CDCl_3_, 500 MHz) *δ:* 8.70 (s, 1H, Qu-2-H), 8.23 (d, *J* = 12.0 Hz, 1H, Cl–Ar–CH=), 7.93 (d, *J* = 8.6 Hz, 1H, Ar–CH=), 7.78–7.85 (m, 3H, Qu-5,7,8-H), 7.47–7.50 (m, 3H, Cl–Ar-2,6-H, Ar-3-H), 7.30–7.39 (m, 5H, Cl–Ar-3,5-H, Ar-4,5-H, Cl–Ar–C=CH), 7.10 (d, *J* = 16.0 Hz, 1H, Ar–C=CH), 6.81 (d, *J* = 14.8 Hz, 1H, Ar-6-H), 2.61 (s, 3H, CH_3_); ^13^C NMR (CDCl_3_, 125 MHz) *δ*: 185.7, 167.4, 154.3, 153.1, 148.1, 147.4, 134.5, 134.1, 133.5, 132.3,131.3, 130.0, 129.8, 129.2, 128.9, 127.3, 127.1, 122.8, 122.6, 121.1, 116.3, 17.8; Anal. Calcd for C_26_H_19_ClN_2_O_2_: C 73.15; H 4.49; N 6.56; Found: C 72.43; H 4.12; N 6.79.

##### (1E,4E)-1-(2-chloro-5-nitrophenyl)-5-(2-((6-methylquinazolin-4-yl)oxy)phenyl)penta-1,4-dien-3-one (**5d**)

Yield: 58.2%; yellow powder; mp: 176–178 °C; IR (KBr, cm^−1^) *ν*: 3445, 1653, 1622, 1576, 1522, 1458, 1348, 1277, 1221, 983; ^1^H NMR (CDCl_3_, 500 MHz) *δ*: 8.68 (s, 1H, Qu-2-H), 8.40 (s, 1H, Cl–Ar-6-H), 8.21 (d, *J* = 15.0 Hz, 1H, Cl–Ar-4-H), 8.10–8.12 (d, *J* = 10.0 Hz, 1H, Qu-8-H), 7.73–7.92 (m, 5H, Cl–Ar–CH=, Qu-5,7-H, Cl–Ar-3-H, Ar–CH=), 7.54–7.57 (m, 2H, Cl–Ar–C=CH, Ar-3-H), 6.91–7.41 (m, 4H, Ar-4,5,6-H, Ar–C=CH), 2.61 (s, 3H, CH_3_); ^13^C NMR (CDCl_3_, 125 MHz) *δ*: 187.8, 179.6, 158.5, 153.3, 151.9, 146.8, 138.1, 136.7, 136.4, 134.6, 132.1, 131.3, 130.7, 130.2, 128.4, 127.8, 126.7, 125.1, 123.6, 122.9, 122.5, 122.2, 116.7, 21.9; Anal. Calcd for C_26_H_18_N_3_O_4_: C 66.18; H 3.84; N 8.90; Found: C 65.81; H 3.66; N 9.30.

##### (1E,4E)-1-(2,4-dichlorophenyl)-5-(2-((6-methylquinazolin-4-yl)oxy)phenyl)penta-1,4-dien-3-one (**5e**)

Yield: 60.5%; yellow powder; mp: 211–214 °C; IR (KBr, cm^−1^) *ν*: 3443, 1655, 1618, 1582, 1499, 1371, 1225, 986; ^1^H NMR (CDCl_3_, 500 MHz) *δ*: 8.68 (s, 1H, Qu-2-H), 8.21 (s, 1H, Qu-5-H), 7.60–7.93 (m, 4H, Qu-7,8-H, Cl–Ar–CH=, Ar–CH=), 7.38–7.43 (m, 4H, Cl–Ar-3-H, Ar-3-H, Cl–Ar-5,6-H), 7.26–7.31 (m, 3H, Ar-4,5-H, Cl–Ar–C=CH), 7.12 (d, *J* = 16.5 Hz, 1H, Ar–C=CH), 6.80 (d, *J* = 16.1 Hz, 1H, Ar-6-H), 2.61 (s, 3H, CH_3_); ^13^C NMR (CDCl_3_, 125 MHz) *δ*: 188.5, 167.1, 153.4, 153.1, 151.4, 142.7, 137.9, 136.6, 136.5, 134.5, 132.3, 131.6, 130.2, 130.1, 128.7, 128.3, 127.6, 127.4, 125.0, 122.7, 121.2, 116.2, 17.7; Anal. Calcd for C_26_H_18_Cl_2_N_2_O_2_: C 67.69; H 3.93; N 6.07; N 7.07; Found: C 67.56; H 3.45; N 5.65.

##### (1E,4E)-1-(2,6-dichlorophenyl)-5-(2-((6-methylquinazolin-4-yl)oxy)phenyl)penta-1,4-dien-3-one (**5f**)

Yield: 55.2%; yellow powder; mp: 187–189 °C; IR (KBr, cm^−1^) *ν*: 3443, 1655, 1618, 1582, 1499, 1333, 1225, 986; ^1^H NMR (CDCl_3_, 500 MHz) *δ*: 8.68 (s, 1H, Qu-2-H), 8.20 (s, 1H, Qu-5-H), 7.89 (d, *J* = 8.5 Hz, 1H, Qu-8-H), 7.80–7.85 (m, 2H, Ar–CH=, Cl–Ar–CH=), 7.73 (d, *J* = 8.8 Hz, 1H, Qu-7-H), 7.52–7.61 (m, 2H, Cl–Ar-3,5-H), 7.39 (m, 1H, Cl–Ar-4-H), 7.24–7.30 (m, 3H, Ar-3,5-H, Ar–C=CH), 7.15 (m, 1H, Ar-4-H), 7.06 (d, *J* = 16.0 Hz, 1H, Cl–Ar–C=CH), 7.00 (d, *J* = 16.5 Hz, 1H, Ar-6-H), 2.60 (s, 3H, CH_3_); ^13^C NMR (CDCl_3_, 125 MHz) *δ*: 188.9, 166.4, 153.4, 151.7, 150.4, 138.4, 137.5, 136.7, 136.5, 135.2, 132.9, 132.3, 131.8, 129.9, 128.9, 128.2, 128.0, 127.9, 127.5, 126.7, 123.6, 122.2, 116.0, 21.9; Anal. Calcd for C_26_H_18_C_l2_N_2_O_2_: C 67.69; H 3.93; N 6.07; Found: C 68.06; H 4.14; N 6.11.

##### 1E,4E)-1-(2,5-dimethoxyphenyl)-5-(2-((6-methylquinazolin-4-yl)oxy)phenyl)penta-1,4-dien-3-one (**5g**)

Yield: 49.6%; yellow powder; mp: 122–123 °C; IR (KBr, cm^−1^) *ν*: 3443, 1653, 1618, 1576, 1497, 1458, 1360, 1223, 1114, 1045; ^1^H NMR (CDCl_3_, 500 MHz) *δ*: 8.68 (s, 1H, Qu-2-H), 8.22 (s, 1H, Qu-5-H), 7.81–7.92 (m, 5H, Qu-7,8-H, Ar–CH=, CH_3_O–Ar–CH=, Ar-3-H), 7.75 (d, *J* = 8.6 Hz, 1H, CH_3_O–Ar–C=CH), 7.51 (m, 1H, Ar-5-H), 7.38 (m, 1H, Ar-4-H), 7.17 (d, *J* = 16.0 Hz, 1H, Ar–C=CH), 6.99 (d, *J* = 2.8 Hz, 1H, Ar-6-H), 6.89–6.94 (m, 2H, CH_3_O–Ar-3,6-H), 6.81 (d, *J* = 2.8 Hz, 1H, CH_3_O–Ar-4-H), 3.76 (s, 6H, 2-OCH_3_), 2.57 (s, 3H, CH_3_); ^13^C NMR (CDCl_3_, 125 MHz) *δ*: 189.3, 166.5, 153.5, 153.4, 153.2, 151.6, 150.4, 138.8, 138.4, 136.6, 136.2, 131.5, 128.4, 128.1, 127.8, 127.1, 126.7, 126.6, 123.5, 122.4, 117.6, 113.2, 112.5, 56.1, 55.8, 21.9; Anal. Calcd for C_28_H_24_N_2_O_4_: C 74.3; H 5.35; N 6.19; Found: C 74.3; H 5.48; N 5.95.

##### (1E,4E)-1-(2-fluorophenyl)-5-(2-((8-methylquinazolin-4-yl)oxy)phenyl)penta-1,4-dien-3-one (**5h**)

Yield: 50.4%; yellow powder; mp: 155–157 °C; IR (KBr, cm^−1^) *ν*: 3445, 1653, 1620, 1582, 1506, 1481, 1398, 1223, 984; ^1^H NMR (CDCl_3_, 500 MHz) *δ*: 8.79 (s, 1H, Qu-2-H), 8.31 (d, *J* = 8.0 Hz, 1H, F–Ar–CH=), 7.77–7.85 (m, 3H, Qu-5,7-H, Ar–CH=), 7.67 (d, *J* = 16.5 Hz, 1H, F–Ar-6-H), 7.59 (m, 1H, Qu-6-H), 7.53 (m, 1H, F–Ar-4-H), 7.29–7.43 (m, 4H, Ar-3,5-H, F–Ar-3,5-H), 7.05–7.14 (m, 3H, Ar-4-H, F–Ar–C=CH, Ar–C=CH), 6.95 (d, *J* = 16.5 Hz, 1H, Ar-6-H), 2.76 (s, 3H, CH_3_); ^13^C NMR (CDCl_3_, 125 MHz) *δ*: 188.8, 167.1, 153.2, 151.7, 151.1, 136.9, 136.6, 136.0, 134.5, 131.9, 131.9, 129.3, 128.4, 128.1, 127.8, 127.8, 127.6, 126.6, 124.5, 123.6, 121.1, 116.4, 17.8; Anal. Calcd for C_25_H_19_FN_2_O_2_: C 76.08; H 4.67; N 6.83; Found: C 75.81; H 4.53; N 7.04.

##### (1E,4E)-1-(2-chlorophenyl)-5-(2-((8-methylquinazolin-4-yl)oxy)phenyl)penta-1,4-dien-3-one (**5i**)

Yield: 41.8%; yellow powder; mp: 152–154 °C; IR (KBr, cm^−1^) *ν*: 3443, 1655, 1616, 1595, 1481, 1406, 1358, 1229, 979; ^1^H NMR (CDCl_3_, 500 MHz) *δ*: 8.79 (s, 1H, Qu-2-H), 8.30 (d, *J* = 8.5 Hz, 1H, Cl–Ar–CH=), 7.96 (d, *J* = 16.5 Hz, 1H, Ar–CH=), 7.76–7.85 (m, 3H, Qu-5,6,7-H), 7.50-7.59 (m, 3H, Ar-3-H, Cl–Ar-3,6-H), 7.38-7.40 (m, 2H, Cl–Ar-4,5-H), 7.29–7.39 (m, 2H, Cl–Ar–C=CH, Ar–C=CH), 7.14–7.25 (m, 2H, Ar-4,5-H), 6.81 (d, *J* = 16.0 Hz, 1H, Ar-6-H), 2.77 (s, 3H, CH_3_); ^13^C NMR (CDCl_3_, 125 MHz) *δ*: 188.7, 167.1, 153.2, 151.7, 151.1, 139.2, 137.2, 136.6, 135.4, 134.6, 131.7, 131.3, 130.3, 128.4, 128.3, 128.1, 127.7, 127.6, 127.1, 126.6, 123.6, 121.1, 116.1, 17.8; Anal. Calcd for C_26_H_19_ClN_2_O_2_: C 73.15; H 4.49; N 6.56; Found: C 73.04; H 4.74; N 6.76%.

##### (1E,4E)-1-(4-chlorophenyl)-5-(2-((8-methylquinazolin-4-yl)oxy)phenyl)penta-1,4-dien-3-one (**5j**)

Yield: 58.6%; yellow powder; mp: 161–163 °C; IR (KBr, cm^−1^) *ν*: 3445, 1647, 1616, 1576, 1481, 1406, 1358, 1227, 937; ^1^H NMR (CDCl_3_, 500 MHz) *δ:* 8.79 (s, 1H, Qu-2-H), 8.20–8.34 (m, 3H, Qu-5,6,7-H), 7.72–7.86 (m, 4H, Ar–CH=, Ar-3-H, Cl–Ar–C=CH, Cl–Ar=CH), 7.52–7.64 (m, 4H, Cl–Ar-2,3,5,6-H), 7.41-7.42 (m, 1H, Ar-5-H), 7.30–7.32 (m, 1H, Ar-4-H), 7.11–7.14 (d, *J* = 15.0 Hz, 1H, Ar-6-H), 6.93–6.96 (d, *J* = 15.0 Hz, 1H, Ar–C=CH), 2.77 (s, 3H, CH_3_); ^13^C NMR (CDCl_3_, 125 MHz) *δ:* 188.6, 167.1, 153.2, 153.1, 151.7, 151.1, 141.9, 136.9, 136.7, 134.6, 131.7, 129.5, 129.2, 128.4, 128.1, 127.6, 127.2, 126.7, 125.8, 123.6, 121.1, 17.8; Anal. Calcd for C_26_H_19_ClN_2_O_2_: C 73.15; H 4.49; N 6.56; Found: C 73.36; H 4.65; N 6.86.

##### (1E,4E)-1-(2-chloro-5-nitrophenyl)-5-(2-((8-methylquinazolin-4-yl)oxy)phenyl)penta-1,4-dien-3-one (**5k**)

Yield: 54.5%; yellow powder; mp: 198–200 °C; IR (KBr, cm^−1^) *ν*: 3420, 1676, 1626, 1560, 1522, 1479, 1402, 1348, 1221, 980; ^1^H NMR (CDCl_3_, 500 MHz) *δ*: 8.78 (s, 1H, Qu-2-H), 8.39 (s, 1H, Cl–Ar-6-H), 8.32 (d, *J* = 8.0 Hz, 1H, Cl–Ar-4-H), 8.13 (d, *J* = 8.3 Hz, 1H, Qu-5-H), 7.76-7.88 (m, 4H, Ar–CH=, Cl–Ar–CH=, Qu-6, 7-H), 7.45–7.59 (m, 3H, Ar-3-H, Cl–Ar-3-H, Cl–Ar–C=CH), 7.23–7.40 (m, 2H, Ar-4,5-H), 7.10 (d, *J* = 12.5 Hz, 1H, Ar–C=CH), 6.93 (d, *J* = 16.0 Hz, 1H, Ar-6-H), 2.75 (s, 3H, CH_3_);^13^C NMR (CDCl_3_, 125 MHz) *δ*: 187.8, 167.1, 153.1, 151.8, 151.0, 146.7, 141.6, 138.2, 136.7, 136.6, 134.6, 132.0, 131.3, 130.1, 128.6, 127.7, 126.9, 126.7, 125.1, 123.7, 122.5, 120.9, 116.1, 17.7; Anal. Calcd for C_26_H_18_ClN_3_O_4_: C 66.18; H 3.84; N 8.90; Found: C 66.30; H 3.84; N 8.86.

##### (1E,4E)-1-(2,4-dichlorophenyl)-5-(2-((8-methylquinazolin-4-yl)oxy)phenyl)penta-1,4-dien-3-one (**5l**)

Yield: 58.6%; yellow powder; mp: 175–178 °C; IR (KBr, cm^−1^) *ν*: 3445, 1653, 1618, 1576, 1481, 1408, 1358, 1229, 984; ^1^H NMR (CDCl_3_, 500 MHz) *δ:* 8.79 (s, 1H, Qu-2-H), 8.30 (d, *J* = 8.0 Hz, 1H, Cl–Ar–CH=), 7.76–7.94 (m, 3H, Ar–CH=, Qu-5,7-H), 7.53–7.57 (m, 2H, Qu-6-H, Cl–Ar-3-H), 7.38–7.47 (m, 3H, Ar-3-H, Cl–Ar-5,6-H), 7.29–7.31 (m, 2H, Cl–Ar-4-H), 7.38–7.41 (m, 2H, Cl–Ar–C=CH, Ar-5-H), 7.15–7.17 (m, 2H, Ar–C=CH, Ar-4H), 6.78 (d, *J* = 16.5 Hz, 1H, Ar-6-H), 2.77 (s, 3H, CH_3_); ^13^C NMR (CDCl_3_, 125 MHz) *δ*: 188.5, 167.1, 153.2, 151.8, 151.1, 139.1, 137.5, 136.7, 135.4, 134.6, 134.1, 133.3, 131.8, 131.7, 129.2, 128.4, 128.0, 127.6, 127.4, 126.7, 125.8, 123.6, 121.0, 116.1, 17.7; Anal. Calcd for C_26_H_18_C_l2_N_2_O_2_: C 67.69; H 3.93; N 6.07; Found: C 67.27; H 4.03; N 5.96%.

##### (1E,4E)-1-(2,6-dichlorophenyl)-5-(2-((8-methylquinazolin-4-yl)oxy)phenyl)penta-1,4-dien-3-one (**5m**)

Yield: 56.1%; yellow powder; mp: 161–163 °C; IR (KBr, cm^−1^) *ν*: 3421, 1676, 1620, 1587, 1481, 1400, 1359, 1225, 984; ^1^H NMR (CDCl_3_, 500 MHz) *δ*: 8.76 (s, 1H, Qu-2-H), 8.28 (d, *J* = 8.5 Hz, 1H, Ar–CH=), 7.73–7.85 (m, 3H, Cl–Ar–CH=, Qu-5,7-H), 7.52–7.59 (m, 3H, Cl–Ar-3,5-H, Qu- 6-H), 7.29–7.41 (m, 4H, Ar-3, 5-H, Cl–Ar-4-H, Cl–Ar–C=CH), 7.16 (m, 1H, Ar-4-H), 7.07 (d, *J* = 16.0 Hz, 1H, Ar–C=CH), 6.98 (d, *J* = 17.0 Hz, 1H, Ar-6-H), 2.75 (s, 3H, CH_3_); ^13^C NMR (CDCl_3_, 125 MHz) *δ*: 188.96, 167.10, 153.15, 151.70, 150.99, 137.59, 136.75, 135.68, 135.17, 134.57, 132.97, 131.85, 129.88, 128.85, 128.36, 127.88, 127.65,127.45, 126.72, 123.60, 121.04, 116.02, 17.79; Anal. Calcd for C_26_H_18_Cl_2_N_2_O_2_ (461): C, 67.69; H, 3.93; N, 6.07; N, 7.07%. Found: 67.36; H, 3.96; N, 5.84%.

##### (1E,4E)-1-(2,5-dimethoxyphenyl)-5-(2-((8-methylquinazolin-4-yl)oxy)phenyl)penta-1,4-dien-3-one (**5n**)

Yield: 43.6%; yellow powder; mp: 176–178 °C; IR (KBr, cm^−1^) *ν*: 3445, 1647, 1616, 1570, 1491, 1373, 1211, 984; ^1^H NMR (CDCl_3_, 500 MHz) *δ*: 8.79 (s, 1H, Qu-2-H), 8.30 (d, *J* = 8.6 Hz, 1H, CH_3_O–Ar–CH=), 7.75–7.92 (m, 4H, Ar–CH=, Qu-5,6,7-H), 7.50–7.59 (m, 2H, Ar-3,5-H), 7.39 (m, 1H, Ar-4-H), 7.15–7.29 (m, 2H, CH_3_O–Ar–C=CH, Ar–C=CH), 6.98 (s, 1H, CH_3_O–Ar-6-H), 6.89–6.93 (m, 2H, Ar-6-H, CH_3_O–Ar-3-H), 6.81 (d, *J* = 8.6 Hz, 1H, CH_3_O–Ar-4-H), 3.77 (s, 6H, 2CH_3_O), 2.76 (s, 3H, CH_3_); ^13^C NMR (CDCl_3_, 125 MHz) *δ*: 189.25, 167.14, 153.57, 153.18, 151.66, 151.03, 138.78, 136.58, 136.25, 134.51, 131.45, 128.41, 128.23, 127.55, 127.24, 126.58, 124.21, 123.54, 121.16, 120.94, 117.61, 116.16, 113.22, 112.47, 56.08, 55.85, 17.75. Anal. Calcd for C_28_H_24_N_2_O_4_ (453): C, 74.32; H, 5.35; N, 6.19; %. Found: C, 74.55; H, 5.68; N, 5.95%.

### Cell culture

Human gastric cancer cell line MGC-803, human prostate cancer cell line PC3, and human breast cancer cell line Bcap-37 and one normal cell line NIH3T3 were obtained from Cell Bank of Type Culture Collection of Chinese Academy of Sciences (Shanghai, China). NIH3T3 was routinely maintained in a DMEM medium, while all the other cell lines were cultured in a 1640 medium. All the cells were grown in the medium supplemented with 10% FBS at 37 °C with 5% CO_2_.

### MTT assay

The growth-inhibitory effects of the test compounds were determined on MGC-803, PC3, Bcap-37, and NIH3T3 cells. All cell types were seeded into 96-well plates at a density of 2 × 10^3^ cells/well 100 μL of the proper culture medium and incubated with increasing concentrations of the compounds at 37 °C under cell culturing conditions. An MTT assay (Roche Molecular Biochemicals, 1465-007) was performed 72 h later according to the instructions provided by Roche. The precipitated formazan crystals were dissolved in SDS, and the absorbance was read at 595 nm with a microplate reader (BIO-RAD, model 680), which is directly proportional to the number of living cells in culture. The experiment was performed in triplicate. The percentage cytotoxicity was calculated using the formula.

$$\% {\text{Cytotoxicity}} = \left[ {\left( {{\text{Control}}_{\text{abs}} - {\text{Blank}}_{\text{abs}} } \right) - \left( {{\text{Test}}_{\text{abs}} - {\text{Blank}}_{\text{abs}} } \right)} \right]/\left( {{\text{Control}}_{\text{abs}} - {\text{Blank}}_{\text{abs}} } \right)\; \times \; 100$$

### AO/EB staining

Cells were seeded in 6-well culture plates at a density of 5 × 10^4^ cells/mL in 0.6 mL of medium and allowed to adhere to the plates overnight. The cells were incubated with different concentrations of compounds or vehicle solution (0.1% DMSO) in a medium containing 10% FBS for 12 h. After the treatment, the cover slip with monolayer cells was inverted on the glass slide with 20 μL of AO/EB stain (100 μg/mL), and finally analyzed for morphological characteristics of cell apoptosis under a fluorescence microscope (Olympus Co., Japan).

### Hoechst 33,258 staining

Cells were seeded in 6-well culture plates at a density of 5 × 10^4^ cells/mL in 0.6 mL of medium and allowed to adhere to the plates overnight. The cells were incubated with different concentrations of compounds or vehicle solution (0.1% DMSO) in a medium containing 10% FBS for 12 h. After the treatment, the cells were fixed with 4% paraformaldehyde for 10 min, followed by incubation with Hoechst 33,258 staining solution (Beyotime) for 5 min and finally analyzed for morphological characteristics of cell apoptosis under a fluorescence microscope (Olympus Co., Japan).

### Flow cytometry analysis

To further quantitative analysis of apoptosis, the cells were washed with PBS, stained with annexinV-FITC and propidium iodide (PI) using the AnnexinV-FITC kit (KeyGEN BioTECH). The cells were then subjected to flow cytometry according to manufacturer’s instructions and the stained cells were analyzed by FACS can flow cytometer (Becton–Dickinson, CA, USA).

### Statistical analysis

All statistical analysis was performed with SPSS Version 19.0. Data was analyzed by one-way ANOVA. Mean separations were performed using the least significant difference method. Each experiment was replicated thrice, and all experiments yielded similar results. Measurements from all the replicates were combined, and treatment effects were analyzed.

## Abbreviations

ADM: adriamycin
AO/EB: acridine orange/ethidium bromide
^13^C NMR: ^13^C nuclear magnetic resonance
DMSO: dimethyl sulfoxide
FCM: flow cytometry
HCPT: 10-hydroxyl camptothecine
^1^H NMR: proton nuclear magnetic resonance
IR: infra-red
MTT: 3-(4,5-dimethylthiazol-2-yl)-2,5-diphenyltetrazolium bromide
TUNEL: terminal deoxynucleotidyl transferase biotin-dUTP nick end labeling
